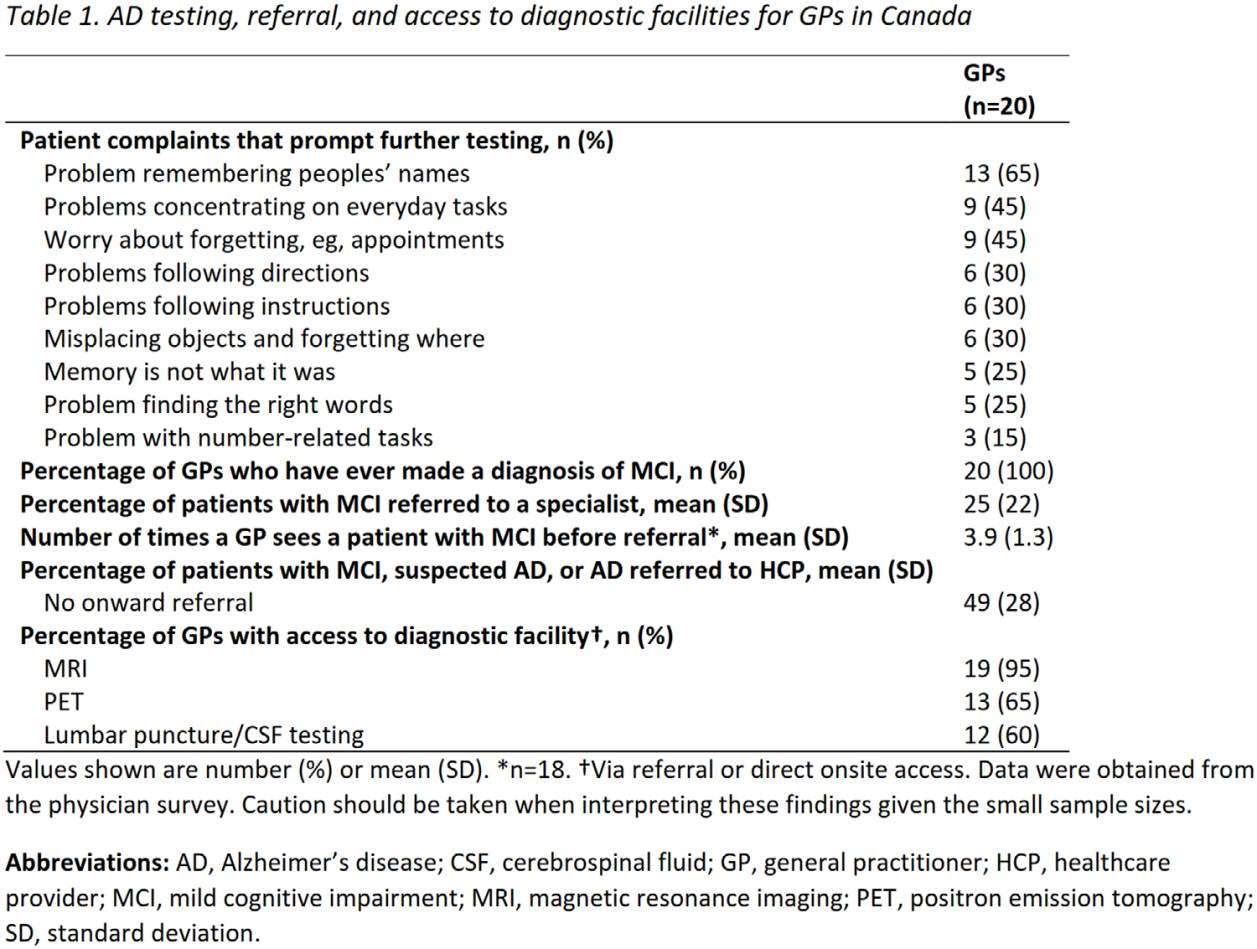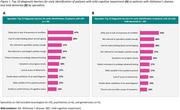# Diagnostic journey and barriers to diagnosis for patients with mild cognitive impairment or dementia due to Alzheimer’s disease in Canada: results from a real‐world survey

**DOI:** 10.1002/alz.095446

**Published:** 2025-01-09

**Authors:** Jennifer Glass, Luc Boulay, Simona Vasileva‐Metiodiev, Chloe Walker, Sarah Cotton, Jean‐Eric Tarride, Robert Laforce, Serge Gauthier

**Affiliations:** ^1^ Eli Lilly Canada Inc., Toronto, ON Canada; ^2^ Eli Lilly & Company, Bracknell, Berkshire United Kingdom; ^3^ Adelphi Real World, Bollington, Cheshire United Kingdom; ^4^ McMaster University, Hamilton, ON Canada; ^5^ Université Laval, Quebec, QC Canada; ^6^ McGill University, Montreal, QC Canada

## Abstract

**Background:**

Alzheimer’s disease (AD) dementia progresses from preclinical brain changes, through mild cognitive impairment (MCI), to AD with dementia. Early diagnosis and confirmation of underlying AD pathology is crucial; however, there is still much to learn about patients’ diagnostic journey. We aimed to describe the diagnostic journey and barriers to diagnosis for patients with MCI or dementia due to AD in Canada.

**Method:**

Data were collected from the Adelphi Real World AD Disease Specific Programme (DSP)™, a cross‐sectional survey of general practitioners (GPs) and specialists in Canada, from March to October 2023. GP surveys covered patient management and referral patterns. Specialists completed a survey capturing attitudes towards diagnosis, advanced testing, and future treatment landscape. Physicians saw ≥5 (GPs) or ≥10 (specialists) patients per week with MCI or dementia/AD. Analyses are descriptive.

**Result:**

The survey was completed by 20 GPs and 30 specialists (19 neurologists, 6 psychiatrists, and 5 geriatricians). GPs reported patient difficulty remembering people’s names (65%) was the patient complaint that most often prompted further testing *(Table 1)*. Other important patient complaints were problems concentrating on everyday tasks (45%) and worry about forgetting things, such as appointments so they have to rely on notes and calendars (45%). GPs reported referring an average of 25±22% of patients with MCI to a specialist after seeing them an average of 3.9±1.3 times. On average, 49±28% of patients with MCI or dementia/AD managed by GPs were not referred to a specialist. Specialists reported the top three barriers to early identification of patients with MCI and patients with mild dementia due to AD were delay due to lack of awareness of the condition (67% and 53%), lack of understanding what constitutes “normal” ageing (63% and 40%), and slow referral (37% and 40%) *(Figure 1)*.

**Conclusion:**

Many patients with MCI or dementia/AD in Canada are not referred by GPs, especially in early‐stage AD. Referrals from GPs were also often delayed, with specialists citing delay due to lack of patient awareness of MCI as the main barrier to early diagnosis. Improving awareness of early AD symptoms and AD pathology, and accelerating access to specialists, are warranted.